# Proteome dynamics during postnatal mouse corpus callosum development

**DOI:** 10.1038/srep45359

**Published:** 2017-03-28

**Authors:** Alexander I. Son, Xiaoqin Fu, Fumikazu Suto, Judy S. Liu, Kazue Hashimoto-Torii, Masaaki Torii

**Affiliations:** 1 Center for Neuroscience Research, Children’s Research Institute, Children’s National Medical Center, Washington, DC 20010, USA; 2 Department of Ultrastructural Research, National Institute of Neuroscience, National Center of Neurology and Psychiatry, Tokyo 187-8502, Japan; 3 Department of Pediatrics, Pharmacology and Physiology, School of Medicine and Health Sciences, The George Washington University, Washington, DC 20052, USA; 4 Department of Neurobiology and Kavli Institute for Neuroscience, School of Medicine, Yale University, New Haven, CT 06510, USA.

## Abstract

Formation of cortical connections requires the precise coordination of numerous discrete phases. This is particularly significant with regard to the corpus callosum, whose development undergoes several dynamic stages including the crossing of axon projections, elimination of exuberant projections, and myelination of established tracts. To comprehensively characterize the molecular events in this dynamic process, we set to determine the distinct temporal expression of proteins regulating the formation of the corpus callosum and their respective developmental functions. Mass spectrometry-based proteomic profiling was performed on early postnatal mouse corpus callosi, for which limited evidence has been obtained previously, using stable isotope of labeled amino acids in mammals (SILAM). The analyzed corpus callosi had distinct proteomic profiles depending on age, indicating rapid progression of specific molecular events during this period. The proteomic profiles were then segregated into five separate clusters, each with distinct trajectories relevant to their intended developmental functions. Our analysis both confirms many previously-identified proteins in aspects of corpus callosum development, and identifies new candidates in understudied areas of development including callosal axon refinement. We present a valuable resource for identifying new proteins integral to corpus callosum development that will provide new insights into the development and diseases afflicting this structure.

The efficient and accurate transmission of cortical signals requires the precise development and maintenance of axon tracts bridging their appropriate locales. The largest collection of these tracts comprises the corpus callosum (CC), a structure bridging the two cortical hemispheres and providing interhemispheric communication essential for cognitive and associative processes^1^,^2^, as well as several higher-order sensory and motor functions^3^,^4^,^5^,^6^,^7^,^8^.

The CC is an intricate structure consisting of cortical axonal projections traversing both cortical hemispheres, along with a complex population of glial cells (astrocytes, oligodendrocytes and microglia) integral to the development and maintenance of this structure. As such, development is an elaborate and multifaceted process requiring the precise regulation of several discrete events. Initial development occurs from the embryonic period, as projections arising mainly from cortical layers II/III and V cross into the midline, traversing into their appropriately targeted regions of the contralateral hemisphere^1^. This initial axon pathfinding and targeting process is followed by the refinement of CC projections, consisting of coordinated preservation and elimination of initially overproduced, or “exuberant,” callosal axons^9^,^10^. This process mainly occurs during the early postnatal period across species including rodents, felines, and primates^11^,^12^,^13^,^14^,^15^. The refinement period is partially overlapped and followed by the maturation of glial cells, subsequently resulting in the formation of myelin sheaths around the established callosal axons^16^.

CC malformation results in serious functional consequences whose effects are linked to abnormalities in specific aspects of this development. Agenesis of the CC (AgCC), in which the structure is completely or partially absent, is associated with various disorders related to severe neurological and cognitive disabilities^1^,^4^,^6^,^17^. Abnormalities in myelin sheath formation within the CC are associated with developmental leukodystrophies such as Alexander disease, Pelizaeus-Merzbacher disease, and various neurometabolic disorders^18^. More subtle anatomical and physiological deficits of this structure have also been commonly reported in several neurodevelopmental disorders^17^,^19^,^20^,^21^,^22^,^23^,^24^,^25^,^26^,^27^, including autism spectrum disorder^28^,^29^,^30^ and schizophrenia^20^,^23^,^31^. As such, clarifying the mechanisms underlying CC development is critical in understanding and addressing these various syndromes.

To date, molecular mechanisms for certain aspects of callosal development have been identified; in particular, factors regulating the early initial axon guidance and much later myelination phases have been well-studied. However, a large chasm of knowledge remains in properly identifying underpinning molecular factors between these two set periods, especially in more subtle aspects of CC development such as that of axon refinement, a process which has been described in several mammalian systems but whose mechanisms remain unknown^32^,^33^,^34^,^35^,^36^,^37^. Filling this gap requires the identification and characterization of CC molecular profiles during these dynamic periods of development.

To characterize the dynamics of molecular profiles of developing CC, we have employed quantitative mass spectrometry-based proteomic profiling of the postnatal mouse CC using the stable isotope labeling in mammals (SILAM) strategy. This database represents a new resource for better understanding both the normal and pathological development of the CC, in particular for its largely unknown refinement process. We demonstrate its value by identifying several protein clusters with unique developmental trajectories, and previously unrecognized protein functional groups potentially involved in CC development during this critical period.

## Results

### Changes of the CC proteome during postnatal development

**Figure 1:**
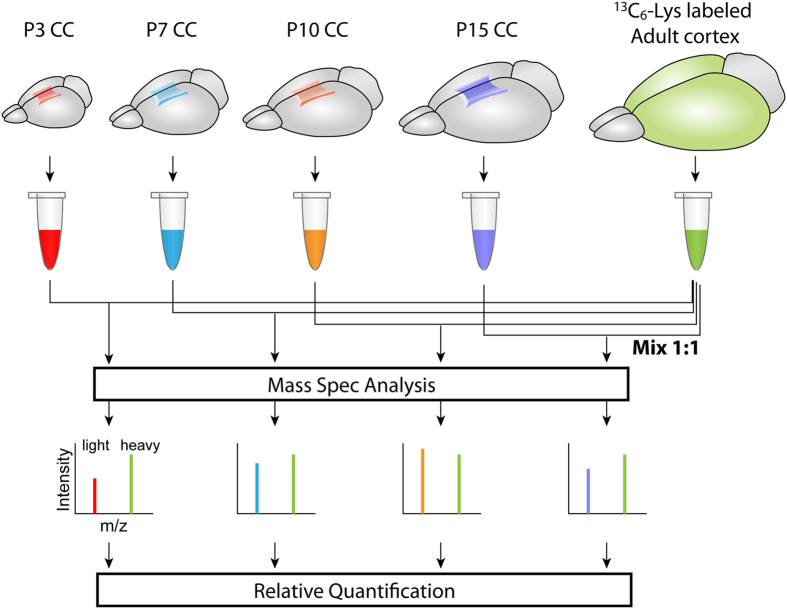
Schematic overview of SILAM. CC tissue of each postnatal age was processed to obtain protein extract. The extract was then mixed at a 1:1 ratio with that similarly obtained from the cerebral cortex of “heavy” ^13^C-lysine-labeled adult C57BL/6 (^13^C-lysine-C57BL/6-SILAC) mice, which serves as the quantitative reference to be compared. The SILAC ratios (heavy/light ratios of peak intensities in the mass spectrum for each peptide of interest) in individual samples reflect the relative protein abundance. The SILAC ratios were log 2 transformed and used for analyses.

We were interested in identifying changes in the proteome of the mouse CC during early postnatal development, focusing especially on the largely understudied period between post midline-crossing of callosal axons and myelination. CC tissue was dissected and collected from CD1 mice at 4 time points [postnatal day 3 (P3), P7, P10, and P15] and was analyzed via quantitative mass spectrometry-based proteomic profiling using the stable isotope labeling of mammals (SILAM) approach (Fig. 1). From analysis with 3 different biological replicates, we identified 2,039 unique proteins (Supplementary Table 1). Density plots of the log2 transformed SILAC ratio showed similar distributions across the samples (Supplementary Fig. 1), confirming the quality of the data.

**Figure 2:**
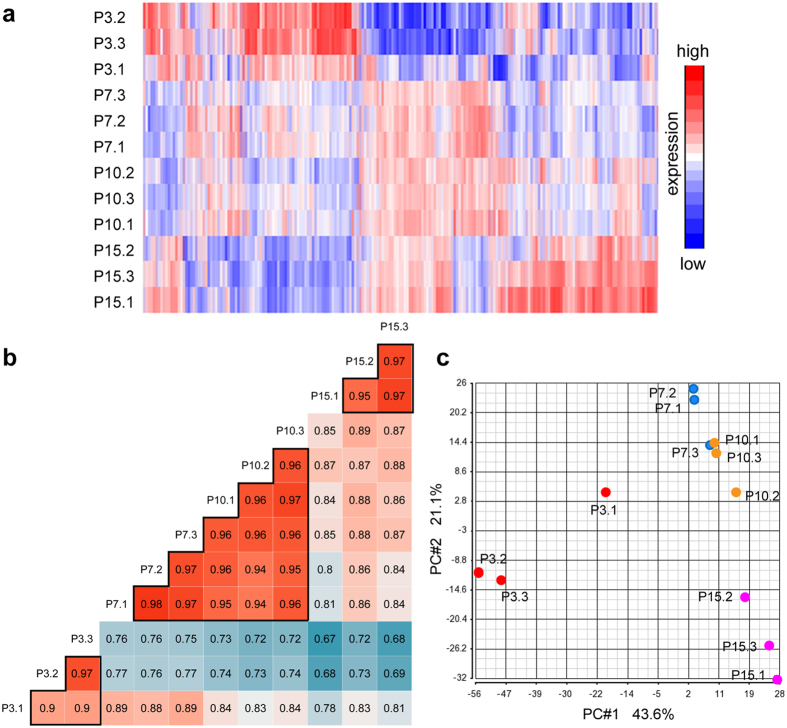
Correlations between samples. (**a**) Protein expression profiles depicted in a heatmap of the log2 transformed SILAC ratio, showing similar expression profiles among the biological replicates of the same age, while differential profiles between tissue samples of different ages. The profiles of P7 and P10 show higher similarities with each other comparing to the profiles of other ages. (**b**) Pair-wise Pearson’s correlation coefficients between the proteomes of samples. The coefficients (values shown for each pair) between biological replicates of the same age are high, while the coefficients between those of different ages are lower, except between P7 and P10, which show high correlations. (**c**) PCAs of the proteomes of samples. P7 and P10 CC samples clustered closely, whereas P3 and P15 CC samples form more isolated clusters.

Protein expression profiles depicted via heatmap showed similar expression profiles among the biological replicates of the same age, while displaying differential profiles between tissue samples of different ages (Fig. 2a). Pearson’s correlation coefficients between biological replicates of the same age were over 0.90, demonstrating excellent technical and biological reproducibility of our proteome analysis (Fig. 2b). Between different ages, correlations were lower, with the correlation coefficients from 0.67 to 0.89; the exception was between P7 and P10, which showed high correlations with the correlation coefficient of over 0.94 (Fig. 2b). This relatively high similarity between P7 and P10 is also observed in the heatmap (Fig. 2a).

To further assess similarities and differences between samples, we performed principal component analysis (PCA). The expression profiles of P7 and P10 CC samples clustered closely, whereas P3 and P15 CC samples clustered alone (Fig. 2c), suggesting that the CC at P7 and P10 are molecularly more similar to each other and distinct from the CC at P3 and P15. These results demonstrate that the proteomic profile of the CC generally differ across time, but remains relatively stable during the period between P7 and P10.

### Temporal dynamics of protein expression in the postnatal mouse CC

**Figure 3:**
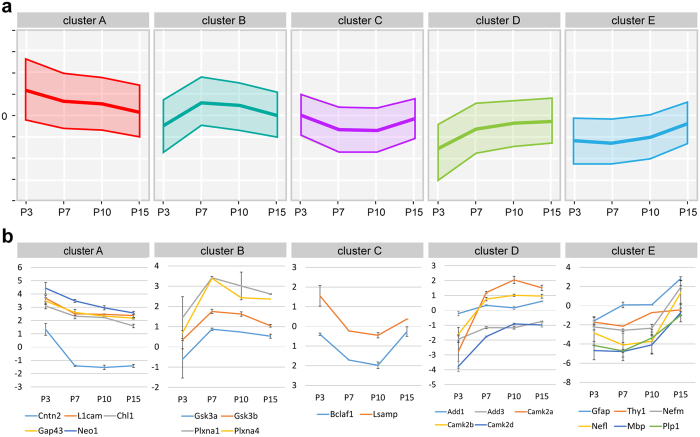
Clustering of proteins expressed in the postnatal CC. (**a**) Developmental trajectories of 5 clusters of proteins with different temporal dynamics of expression in the postnatal CC. The middle and upper/lower line in each graph represent the centroid (mean level of log2 transformed SILAC ratios) for each cluster and the standard deviation. (**b**) Developmental trajectories of representative proteins in each clusters. The centroid graphs show mean expression levels of proteins. Error bars represent the standard deviation.

After excluding proteins for which the expression data for any of the 4 time points were unavailable, we performed k-means clustering of time-course profiles of these 1,658 proteins. We then classified the resulting profiles into 5 distinct clusters with the use of the R package *cclust*^38^ to determine the optimal number of clusters (Supplementary Table 2). These clusters contain 439 (cluster A), 316 (cluster B), 206 (cluster C), 440 (cluster D) and 257 (cluster E) proteins, respectively, with each cluster showing a unique developmental trajectory of expression (Fig. 3a).

**Figure 4:**
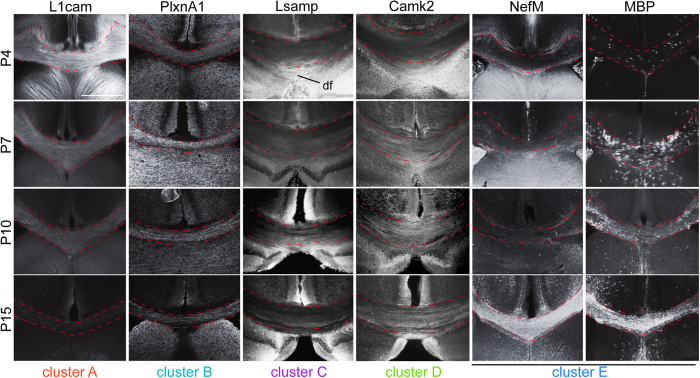
Temporal expression patterns of identified proteins in each cluster in the postnatal CC. Immunohistochemistry for representative proteins with unique temporal dynamics of expression in the postnatal CC (demarcated with broken lines). The expression of L1cam in the CC decreases from P4 through P15, while PlxnA1 shows its peak at P7. The expression of Lsamp in the CC decreases between P4 and P7, but then is increases by P15. The expression of Camk2 is low at P4, but then increases and by P7 and is maintained in later stages. The expression of NefM and MAB in the CC increases as the structure matures from P4 through P15. df: dorsal fornix.

To validate the robustness of our SILAM-based proteomics and time-course protein profiling, we performed immunohistochemistry for several representative proteins expressed in neuronal and/or glial cells: L1cam (cluster A), PlxnA1 (cluster B), Lsamp (cluster C), Camk2 (cluster D), MBP and Nefm (cluster E) at P4, P7, P10 and P15. The labeling pattern of these proteins showed specific temporal patterns in the CC (Fig. 4) consistent with the clusters they belong (Fig. 3b), confirming the validity of our temporal profiling of SILAC-based quantitative proteome. This provides a solid foundation for further characterization of CC development using the obtained time-course profiles.

### Protein functions relevant to unique developmental trajectories

The proteins in each of the clusters have distinct and unique developmental trajectories, indicating noticeable features of the developing CC at distinct postnatal ages. Cluster A, which consists of proteins whose expression decrease from the highest at P3, includes many proteins involved in axon growth and guidance, such as Cntn2, ChL1, Gap43, L1cam, and Neo1^39^,^40^ (Fig. 3b), consistent with the growth of callosal axons from layers II-III through the CC during the early postnatal period^41^,^42^. In contrast, cluster E, which consists of proteins whose expression strongly increase between P10 and P15, includes the astrocyte marker GFAP; late neuronal markers Thy1, Nefm and Nefl; and myelin markers MAB and Plp1, consistent with the roles of these proteins in axonal/glial maturation and myelination of the CC.

Comparing to clusters A and E, which show overall expression changes in proportion to the progress of CC development, clusters B, C, and D show unique patterns of temporal changes at P7 and/or P10 (Fig. 3b), suggesting unique roles of proteins in these clusters in CC development. Cluster B shows temporal increase in expression during P7 through P10. In this cluster, we found Gsk3b, which is involved in cellular division, proliferation, motility and survival, as well as axon guidance and degeneration^43^,^44^. We have also identified PlxnA1 and PlxnA4, members of plexin family molecules that have been shown to play roles in neurite pruning in other systems^34^. Cluster C, on the other hand, shows temporally low expression during P7 to P10. In this cluster, we had identified Lsamp and Bclaf1. Lsamp is involved in axon growth^45^ and also serves as a negative regulator of myelination^46^, while Bclaf1 binds to bcl-related proteins, and induce apoptosis and autophagy^47^,^48^. These proteins may play important roles in the developmental refinement of the CC^9^,^10^.

Cluster D includes proteins whose expression show strong increase between P3 and P7, and maintained afterwards. In this cluster, we found Camk2a, b, and d, isoforms of Camk2 that play roles in growth and guidance of postcrossing callosal axons^49^; and the adducin proteins (Add1 and 3), which are known to regulate actin cytoskeleton and be involved in axon diameter maintenance^50^,^51^. Thus the molecules that play specific roles in post-crossing of callosal axons, particularly their maturation and maintenance, are likely enriched in this cluster.

We also examined whether proteins associated with human ACC are included in these clusters. We referred the ACC-associated genes in a recent comprehensive review of clinical and genetic findings on CC development syndromes by Edwards *et al*.^16^. Several ACC-associated genes were identified in each cluster with the exception of cluster C (in which, coincidentally, the least number of proteins were included) (Table 1), suggesting that ACC-associated proteins play important roles in various distinct phases in CC development.

**Figure 5:**
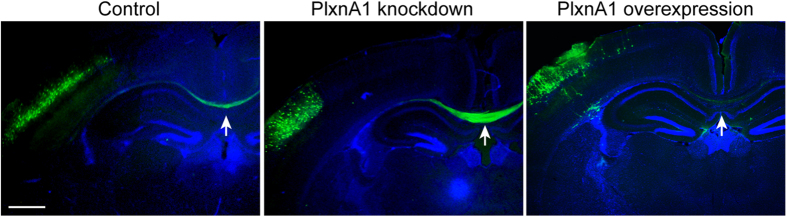
*In vivo* functional analysis of PlxnA1 via *in utero* electroporation. Wild-type mouse brains electroporated at E15.5 and visualized at P15. PlxnA1 knockdown reveals more crossed callosal axons compared to the Control (control shRNA), while PlxnA1 overexpression shows a visible reduction (see arrows) [controls for overexpression (electroporation with an empty vector; data not shown) show the same results as the shRNA controls]. Cortical neuron migration and axon pathfinding appear grossly unaltered by PlxnA1 manipulation.

To assess the value of these molecular profiles to decipher novel players controlling the CC development such as largely unknown process of CC refinement, we performed a brief functional assay on the potential role of one of the identified proteins, PlxnA1, in CC development. We chose to look at PlxnA1 as it has been previously identified as a schizophrenia susceptibility gene^52^,^53^,^54^, a disorder which has extensively been associated with malformations of the CC^6^,^55^,^56^,^57^. In addition, a recent study has shown that polymorphisms in *PlxnA* genes including *PlxnA1* mediate the developmental trajectory of human corpus callosum microstructure^58^. This was accomplished by utilizing *in utero* electroporation at E15.5 to transfer plasmids for knocking down or overexpressing PlxnA1 in callosal projection neurons in layers II/III. Plasmids that were electroporated include a short hairpin RNA (shRNA) of *PlxnA1*^59^ (*PlxnA1* knockdown), cDNA of mouse full-length PlxnA1^60^ (PlxnA1 overexpression), and their corresponding control constructs. These constructs were co-electroporated with a Green Fluorescent Protein (GFP)-expression plasmid to visualize transfected neurons and their callosal projections. Observations at P15 revealed that brains which received *PlxnA1* shRNA appeared to contain more GFP-labeled axons within the CC comparing to control-electroporated brains with similar number of electroporated neurons in layers II/III (n = 5 each). Conversely, brains which received PlxnA1 overexpression appeared to contain much less GFP-labeled axons within the CC compared to the control (n = 4 each) (Fig. 5). We did not observe any obvious abnormal targets within these brains. These results suggest that PlxnA1 may play a critical role for adjusting axonal numbers to be formed or maintained in CC development (e.g. through a mechanisms such as neurite pruning reported in other systems^34^). Although further studies, including quantitative analysis, are essential to define the role of PlxnA1 in CC development, these data demonstrate a functional method of identifying the physical functions of the proteins identified within our screen.

### Functional annotations enriched in association with unique developmental trajectories

**Figure 6:**
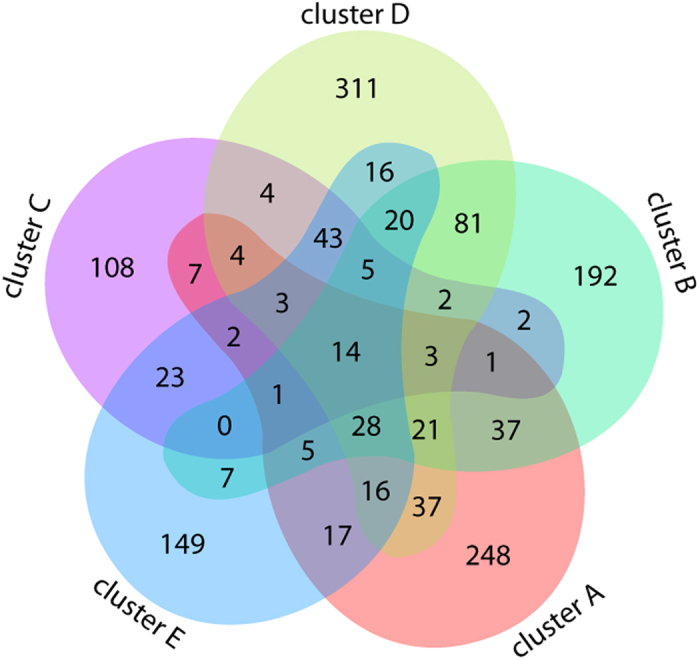
Common and unique GO terms between clusters. The enriched GO terms (EASE Score <0.05) were examined for common and unique terms using Venn diagram.

To determine the biological context behind each clusters in an unbiased manner, we next employed functional annotation analysis using the Database for Annotation, Visualization and Integrated Discovery (DAVID)^61^, which provides the ranking of gene ontology (GO) annotations enriched in each cluster (Supplementary Table 3, with Enrichment Score). Multiple comparisons of the enriched GO terms obtained for proteins in each cluster [EASE Score (a modified Fisher Exact P-Value)^62^ cut off of 0.05] (Fig. 6, Supplementary Table 4) showed only 14 common GO terms between all clusters out of 1,407 overall number of unique elements. In contrast, significant numbers of GO terms were uniquely specified to one cluster only: 248, 192, 108, 311 and 149 terms were uniquely specified to clusters A through E, respectively (Fig. 6), validating that each individual cluster associated with a unique developmental trajectory pattern consists of proteins with specific functional/molecular characteristics. Relatively higher similarities were found between clusters B and D, and between clusters C and E: 174 GO terms overlap between clusters B (41.5% of 419 terms) and D (28.6% of 608 terms), and 91 GO terms overlap between clusters C (41.0% of 222 terms) and E (26.1% of 349 terms).

**Figure 7:**
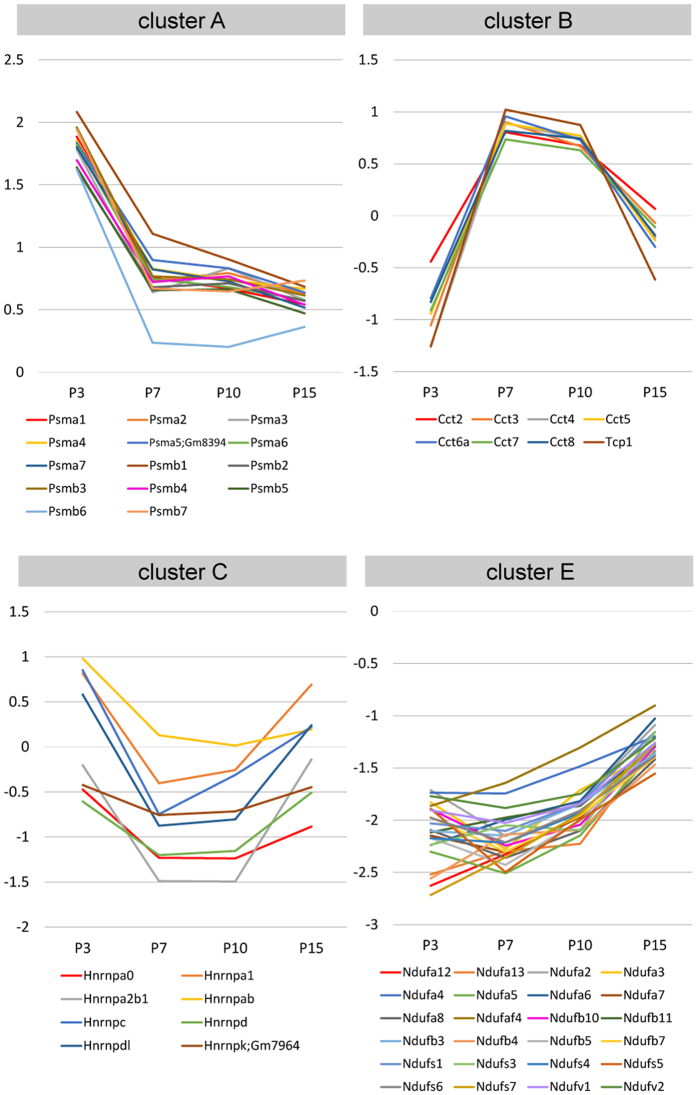
Developmental trajectories of unique groups of proteins enriched in specific clusters. Developmental trajectories of proteasome subunits (Psma1-7 and Psmb1-7), Tcp1 and chaperonin containing Tcp1 subunits (Cct2-8 and Tcp1), heterogeneous nuclear ribonucleoproteins (Hnrnp proteins) and NADH:ubiquinone oxidoreductase subunits (Ndufa, Ndufb, Ndufs and Ndufv) depicted by centroid graphs, showing unique patterns of temporal dynamics that are highly conserved within each protein group.

The obtained annotations were further processed for functional annotation clustering using DAVID to group similar annotations together (Supplementary Table 5). In cluster A, proteins associated with the GO annotations “proteasome/protease/peptidase” (e.g., specific proteasome subunit: Psma1-7 and Psmb1-7) were highly enriched and showed very similar expression trajectories (Fig. 7), indicating rapid turnover of proteins during this period. In this cluster, we also found that proteins associated with “axon/neuronal projection,” “microtubule cytoskeleton,” and “intracellular protein transport” were highly enriched, consistent with extensive/continuous axonal growth and guidance during the early postnatal period.

In cluster B, strong enrichment of proteins associated with “chaperonin/chaperon” and “protein folding” (e.g., Tcp1 and chaperonin containing Tcp1 subunit: Cct2-8 and Tcp1) was observed (Fig. 7). Those associated with “nucleotide-binding” were also observed, suggesting the critical role of molecular chaperons in correct folding of proteins during specific period during P7 through P10.

In cluster C, an enrichment of proteins associated with “RNA splicing/processing” (e.g., heterogeneous nuclear ribonucleoproteins: Hnrnp proteins) was observed (Fig. 7), indicating temporal and transient decrease of these post-transcriptional processing processes in axons and glial cells in the CC around P7 through P10.

In cluster D, enrichments of proteins associated with “nucleotide-binding”, “mitochondrion” and “translation” were observed, again suggesting increasing energy requirement and translation during axonal and glial maturation.

In cluster E, we found very strong enrichment of proteins associated with “mitochondrial membrane” and “respiratory chain/electron transport chain” [e.g., NADH:ubiquinone oxidoreductase subunit (also called NADH dehydrogenase (ubiquinone) subunit): Ndufa, Ndufb, Ndufs and Ndufv] proteins, with very similar expression trajectories (Fig. 7), suggesting increase in energy requirement in association with neuronal and glial maturation. Proteins associated with “ribosomal protein” were also enriched, suggesting an increased requirement of translational machinery locally in maturing axons and/or in maturing glial cells within CC.

## Discussion

Here, we profiled the proteome of the postnatal mouse CC by SILAM-mediated quantitative mass spectrometry across multiple time points. This strategy is of particular strength in performing such a developmental screen in this cortical structure; while other high-throughput transcriptome-profiling technologies address transcriptional events involved in the development of various brain structures, they are not best suited for addressing gene expression in callosal axons for which neuronal cell bodies sit in the cortex. Our analysis revealed that among the four time points that we had observed, the CC proteomes are substantially different between three distinct stages: an early phase (reflected at P3), an intermediate phase (reflected by similar proteome profiles at P7 and P10), and a later phase (reflected at P15) (Fig. 2). One possibility for this segregation is that this may be a reflection of the progression of major neurodevelopmental processes in the CC. For example, the crossing of callosal axons from layers II/III occurs around P3, while myelination begins around P10^41^,^42^,^63^. Future work will be required to confirm whether the component proteins observed at these phases are stable as a result of balanced and discrete neurodevelopmental processes, or are reflective of other aspects of corpus callosum development.

Clustering analysis of time-course profiles using our database categorized the proteins with their unique developmental trajectories relevant to their functions into distinct clusters (Fig. 3). Many proteins with specific roles in axon growth and guidance were included in the cluster that shows highest expression at P3 (cluster A), while proteins involved in axonal/glial maturation and myelination were included in the cluster that shows strong expression increase after P10 (cluster E). The clustering analysis also revealed that many proteins show unique temporal changes in their expression during the period between P7 and P10 (clusters B, C and D). Of note, these proteins include those with functions in cell death or survival, autophagy, axon structure maintenance, neurite pruning and degeneration, and myelination. The results suggest that this specific period is a critical transitional phase for the CC from its initial growing phase to its maturation and maintenance phase through extensive refinement, which includes coordinated axon preservation and elimination, and contribution of glial cells^9^,^10^.

One consideration we have not made for this study has been gender differences of the corpus callosum, as both male and female corpus callosi were utilized for our proteomics screen at each of the time points. Previous literature has identified effects of sexual dimorphism on the white matter^64^. Future studies segregating male and female corpus callosi may prove to be informative in deciphering these differences.

An important consideration of this data is the timing of callosal axon populations, as midline crossing of deeper cortical layer axons (projections from mostly layer V) occurs embryonically while that of upper cortical layer axons (projections from layers II/III and the major population of callosal axons) occur at P2-3^41^,^42^,^65^. Myelination of the corpus callosum begins at approximately P10^63^, although whether this event occurs sequentially based on specific axonal populations or occurs concurrently remains unclear. Based on these known events and our own timed proteome data, we may be able to infer some insight as to which layered cortical projections are expressing these proteins at specific temporal windows. For instance, axon growth and guidance related proteins that are observed at P3 likely reflect predominantly axonal projections from cortical layer II/III, while myelination-related proteins observed at P15 are likely reflecting expression in both layers II/III and V. For proteins regulating axons elimination and maintenance during CC refinement, modification of layer V projections may be occurring earlier than those for layers II/III. This raises the possibility that axon refinement proteins identified at P3 may be reflective of layer V projections while those identified at P7 and/or P10 may reflect layer II/III projections. Future studies deciphering proteome expression for specific projections of precise cortical layers over distinct temporal time points may provide powerful insight into the developmental changes undergoing these individual layers.

This data set can be utilized for analyzing the temporal expressions of proteins linked to neuropsychiatric disorders associated with CC abnormalities, potentially identifying key impacts of these proteins during their expression during CC development. Our initial analysis has identified several ACC-associated genes in each cluster (Table 1), which is important given that deficiencies in their stage-specific functions may cause specific defects in the CC, thereby contributing to unique disorders with CC abnormalities^66^,^67^. Known diseases caused by mutations in the identified genes include: L1 syndrome by mutation in *L1cam*^68^,^69^, Warburg micro syndrome by mutations in *Rab18*^70^, and Miller-Dieker syndrome by deletion of *Ywhae*^71^,^72^ (cluster A); Malan syndrome (Sotos syndrome 2) and Marshall-Smith syndrome by *Nfix* haploinsufficiency^73^,^74^,^75^,^76^, and FG syndrome by mutation in *Flna*^77^,^78^ (cluster B); Fumarase deficiency by mutations in *FH*^79^,^80^,^81^ (cluster D); Pyrvate dehydrogenase deficiency by mutations in *pdha1* or *pdhb*^82^ (cluster E). In addition, other identified genes associated with disorders affecting the CC have been identified, such as *PlxnA1* and its link with schizophrenia^52^,^53^,^54^. Many of these diseases are uniquely associated with mental retardation, cognitive impairment and intellectual disability.

Following functional annotation analysis using the DAVID further underlines the importance of our datasets in identifying biological processes and proteins associated with unique mechanisms in CC development. We identified many functional groups with specific GO annotations highly enriched in each cluster. In addition to identifying the enrichment of GO annotations “axon/neuronal projection,” “microtubule cytoskeleton,” and “intracellular protein transport” in cluster A (as expected, given that axon growth/guidance occurs first in CC development), we have also identified new functional groups enriched in other clusters. For example, we found enrichment of chaperonin containing Tcp1 subunits (Cct2-8, and Tcp1) in cluster B, which shows a unique pattern of high expression at P7 and P10 (Fig. 3). It has been shown that the most abundant clients of Cct are the actin and tubulin families of cytoskeletal proteins^83^,^84^,^85^ suggesting that proper folding of actin and tubulin molecules are highly essential during this specific period.

Cluster C, which shows a pattern of temporal/transient low expression during P7-P10, showed strong enrichment of heterogeneous nuclear ribonucleoproteins, Hnrnp proteins (Fig. 7). Mutations and functional abnormalities of Hnrnps are linked to neurodegenerative disorders including amyotrophic lateral sclerosis (ALS)^86^,^87^,^88^,^89^ which is associated with the CC degeneration^90^,^91^. Reduced expression of these proteins may be a part of the mechanism to eliminate exuberant callosal axons via developmental degeneration machinery for CC refinement.

Strong enrichment of NADH:ubiquinone oxidoreductase (respiratory complex I) subunits, Ndufa, Ndufb, Ndufs and Ndufv was observed in cluster E, which shows an expression pattern of significant increase after P10 (Fig. 7). These proteins are associated with GO annotations “Parkinson’s disease,” “Huntington’s disease,” and “Alzheimer’s disease,” as dysfunctions of respiratory complex I are linked to an increasing number of neuromuscular and neurodegenerative diseases, as well as to oxidative stress and ageing^92^. Corpus callosal atrophy or shape changes has been reported in premanifest and early Huntington’s disease and Alzheimer’s disease^93^,^94^,^95^,^96^. As such, dysfunction of these proteins may critically contribute to the degeneration of the CC in these neurodegenerative disorders.

Collectively, the data from this study is a valuable resource for understanding the CC development. In particular, our dataset may be used to distinguish previously unidentified proteins important for distinct aspects of CC development and maintenance, including proteins regulating the largely underexplored period of CC refinement. This will also help to decipher molecular disturbances in genetically and environmentally induced neuropsychiatric disorders associated with CC abnormalities.

## Methods

### Animal handling

All animals were handled according to protocols approved by the Institutional Animal Care and Use Committees of the Children’s National Medical Center and National Institute of Neuroscience, National Center of Neurology and Psychiatry. All methods were performed in accordance with the relevant guidelines and regulations. Embryonic day 0.5 (E0.5) and postnatal day 0 (P0) were designated as noon of the day on which the presence of a vaginal plug was observed and the day of birth, respectively.

### Generation of ^13^C-lysine-C57BL/6-SILAC mice

The generation of ^13^C-lysine-C57BL/6-SILAC (stable isotope labeling with amino acids) mice has been previously described^97^,^98^,^99^. Briefly, pregnant female mice were fed mouse feed containing ^13^C-lysine (99%; Cambridge Isotope Laboratories; Andover, MA) continuously until the F2 generation litters were born. Cortical brain tissue from male and female F2-generation labeled mice was collected.

### Tissue collection and sample processing

For collection of corpus callosi tissue, untreated CD1 mouse (Charles River, Wilmington, MA) brains at postnatal ages 3 (P3), P7, P10, and P15 (1 male, 2 females at each stage) were dissected from the skull and placed dorsal surface down on a nitrocellulose membrane. Coronal brain sections were cut at 1 mm intervals with a Mcllwain Tissue Chopper. Corpus callosi were isolated from these sections, individually flash frozen, and stored at −80 °C until mass spectrometry tissue processing. For collection of ^13^C-lysine-C57BL/6-SILAC cortex, adult treated mouse brains were removed from the skull. Mouse cortices were removed, and tissue was subsequently flash frozen and stored at −80 °C until mass spectrometry tissue processing.

Fast-frozen CD1 corpus callosi and ^13^C-lysine-C57BL/6-SILAC cortex was processed in lysis buffer (20 mM Tris-Cl, 1% Triton X-100, 150 mM NaCl, 10% glycerol, 5 mM Ethylenediaminetetraacetic (EDTA), 1x proteinase and phosphatase inhibitor cocktail (Roche, Basel, Switzerland) on ice for 30 min, followed by centrifugation at 13,000 rpm for 15 min at 4 °C, after which the supernatant of lysates were collected.

Aliquots of protein extracts from the respective CD1 corpus callosi were mixed with labeled adult C57BL/6 cortex at a 1:1 ratio (30 µg each), and detergent was subsequently removed using Pierce Detergent Removal Spin Columns (Thermo Fischer Scientific, Waltham, MA) as per the manufacturer’s instructions. Protein samples were then digested using the SMART digest system (Thermo Fischer Scientific) and were incubated at 70 °C at 1400 RPM for 2 hours. Samples were separated into 8 different fractions using the Pierce High pH Reversed-Phase Peptide Fractionation kit (Thermo Fisher Scientific) as per the manufacturer’s instructions, and subsequent protein samples were subject to mass spectrometry analysis.

### Mass Spectrometry

The mass spectrometry (MS) procedure has been described previously. Fractionated peptides were injected via an autosampler (6 μl) and loaded onto a Symmetry C18 trap column (5 μm, 300 μm inner diameter × 23 mm; Waters, Milford, MA) for 10 min at a flow rate of 10 μl/min with 0.1% formic acid. Samples were subsequently separated with a C18 reversed-phase column (3.5 μm, 75 μm × 15 cm, LC Packings, Sunnyvale, CA) at a flow rate of 250 nl/min with a Nano-HPLC system (Eksigent, Dublin, CA). The mobile phases consisted of water with 0.1% formic acid and 90% acetonitrile. A 65-min linear gradient from 5% to 40% acetonitrile was employed. The eluted peptides were then introduced into the mass spectrometer via a 20-mm-inner-diameter, 10- μm silica tip (New Objective) adapted to a nanoelectrospray source (Thermo Fisher Scientific). Spray voltage was set at 1.4 kV, and the heated capillary was set at 200 °C. The LTQ-Orbitrap-XL (Thermo Fisher Scientific) was operated in a data-dependent mode with dynamic exclusion, in which one cycle of experiments consisted of a full mass spectrometry in the Orbitrap (300–2000 m/z) survey scan at resolution of 30,000. Five subsequent tandem mass spectrometry (MS/MS) scans in the linear trap quadrupole of the most intense peaks in centroid mode using collision-induced dissociation with the collision gas (helium) and normalized collision energy value was set at 35%.

### Data processing

Mass spectral data was uploaded in to the IP2 software (version 1.01), which was then used to identify and quantify proteins. Mass spectral files were searched against the UniProt mouse database (UniProt release-2010_11; 16,333 entries) and indexed (fully tryptic, 300–4000 mass range, and two missed cleavages and for potential modification of oxidized methionine (15.99492 Da) and heavy Lys (6.020 Da) using the SEQUEST algorithm in the BioWorks software package (version 3.1, Thermo Finnigan, San Jose, CA). Mass tolerances were set at an error of 50 ppm for MS and 1 Da error for MS/MS). Data were filtered by setting the protein false discovery rate at less than 1%, and proteins identified by at least two unique peptides were retained for subsequent analysis. The IP2 platform equipped with Census software (version 1.77) was utilized to determine the ratios of unlabeled-to-labeled peptides using an extracted chromatogram approach. Quantitative data were filtered based on a determinant value of 0.5 and an outlier *p* value of 0.1.

### Bioinformatics analyses

To confirm the quality of our data, density plots of protein expression values (log2 transformed SILAC ratios) were generated by MeV software^100^. The relationships between samples were assessed by generating a heatmap and performing principal component analysis (PCA) for protein expression values using Partek Genomics Suite software. Pairwise Pearson’s correlation coefficients were calculated on the expression values (log2 transformed SILAC ratios) and visualized using an R package *ggcorr*.

For k-means clustering, the expression values (log2 transformed SILAC ratios) were applied to an R package *cclust*^38^ to determine the optimal number of clusters. K-means clustering was then performed using MeV.

Functional annotation analysis and annotation clustering was performed for each protein cluster using The Database for Annotation, Visualization and Integrated Discovery (DAVID). Databases used were Gene Ontology Biological Process (GO-BP), Molecular Function (GO-MF), Cellular Compartment (GO-CC), Interpro, KEGG, Smart, PIR Superfamily, Swiss Prot PIR keywords, Up Seq Feature, BioCarta and NCBI’s COG database. The enriched GO terms (p < 0.05) were examined for common and unique terms, and visualized with Venn diagram using an online tool at the Bioinformatics & Evolutionary Genomics website (http://bioinformatics.psb.ugent.be/cgi-bin/liste/Venn/calculate_venn.htpl).

### Immunohistochemistry

Brains were fixed with 2–4% paraformaldehyde in phosphate buffered saline for 3 hours to overnight. 70 μm thick coronal vibratome slices or 12–16 μm coronal cryosections were collected. Immunohistochemistry was performed as described previously (n > 5 brains per antibody)^101^,^102^,^103^. The following primary antibodies were used; mouse monoclonal anti-CaMKII (1:50; Santa Cruz Biotechnology, Dallas, TX), anti-MBP (1:500; Abcam, Cambridge, MA) and anti-Neuroflament-165 kDa (1:500; DSHB, Iowa City, IA), rat monoclonal anti-L1 (1:1000; EMD Millipore, Billerica, MA) and anti-Lsamp (1: 500; DSHB), and rabbit polyclonal anti-PlxnA1 (1:200; see below) antibodies.

The rabbit serum antibody against mouse PlxnA1 was produced by subcutaneous injection of the recombinant proteins for the sema domain of PlxnA1 (aa:23–595) fused with human IgG-Fc domain and 6xHis-tag (PlxnA1SD-Fc-His6; 200 μg/400 μl for each shot), 5 times at 2-weeks intervals. The antibody was affinity purified with the recombinant proteins conjugated to Affi-Gel 10 (BioRad). The specificity of the antibody was confirmed by immunohistochemistry with the brain sections of E16.5 *PlxnA1* wild-type and knockout mice (Fig. S2).

The slices and sections were nuclear counterstained with TO-PRO3 or DAPI (Molecular Probes, Eugene, OR) and photographed with a LSM510 (Carl Zeiss, Oberkochen, Germany) or BZ-X710 (KEYENCE, Osaka, Japan).

### *In utero* electroporation

Following constructs were used: *PlxnA1* shRNA and control (encoding *PlxnA1* shRNA with a single-base mutation) plasmids^59^ (5 μg/μl), PlxnA1 overexpression and control (an empty vector) plasmids^60^ (2 μg/μl) and pCAG-GFP^101^,^102^ (1 μg/μl). E15.5 pregnant CD-1 mice were anesthetized with ketamine/xylazine (100/10 mg/kg), after which the uterine horns were exposed. DNA was injected via a pulled glass pipette into the lateral ventricles of the targeted embryo, after which electrodes were placed onto both sides of the head parallel to the sagittal plane. An electrical current (five 50 ms pulses of 40 V at 950 ms intervals) was then passed to drive DNA into the parietal cortical areas using an ECM 830 Square Wave Electroporator (Harvard Apparatus, Holliston, MA). After electorporation, the uterine horns were returned and the mouse was allowed to recover and give birth normally. Animals positive for GFP expression in the parietal cortical areas at the similar levels were sacrificed at P15, and fixed brains were sectioned into 70 μm vibratome slices. Slices were nuclear counterstained with DAPI and imaged with a BX-61 microscope (Olympus, Tokyo, Japan).

### Data deposition

The mass spectrometry proteomics data have been deposited in ProteomeXchange Consortium via PRIDE^104^ partner repository with the dataset identifier PXD005271.

## Additional Information

**How to cite this article:** Son, A. I. *et al*. Proteome dynamics during postnatal mouse corpus callosum development. *Sci. Rep.* **7**, 45359; doi: 10.1038/srep45359 (2017).

**Publisher's note:** Springer Nature remains neutral with regard to jurisdictional claims in published maps and institutional affiliations.

## Supplementary Material

Supplementary Information

Supplementary Table S1

Supplementary Table S2

Supplementary Table S3

Supplementary Table S4

Supplementary Table S5

## Figures and Tables

**Table 1:** List of genes associated with human ACC for the proteins in each cluster.

cluster A	cluster B	cluster C	cluster D	cluster E
*ampd2*	*arhgef7*		*cplx1*	*letm1*
*flna*	*cask*		*dmd*	*mecp2*
*igbp1*	*dis3l2*		*fh*	*pdha1*
*l1cam*	*gdi1*		*tubb2a*	*pdhb*
*marcks*	*gsk3b*			
*ncam1*	*nfia*			
*rab18*	*nfix*			
*ranbp1*	*tubb2b*			
*ywhae*	*tubb3*			

List of genes associated with human ACC for proteins identified in each of the 5 clusters. No genes were found in the smallest cluster C.
