# Supplementary material for: Proteome dynamics during postnatal mouse corpus callosum development
Source: Sci Rep. 2017 Mar 28;7:45359. doi: 10.1038/srep45359 (PMC5368975; doi:10.1038/srep45359)
Supplement: Supplementary Information [file srep45359-s1.pdf]

Supplementary Information

Proteome dynamics during postnatal mouse corpus callosum development

Alexander I. Son[1], Xiaoqin Fu[1], Fumikazu Suto[2], Judy S. Liu[1,3], Kazue Hashimoto-Torii[1,3,4] and Masaaki Torii[1,3,4,*]

[1]Center for Neuroscience Research, Children's Research Institute, Children's National Medical Center, Washington, DC 20010

[2]Department of Ultrastructural Research, National Institute of Neuroscience, National Center of Neurology and Psychiatry, Tokyo 187-8502, Japan.

[3]Department of Pediatrics, Pharmacology and Physiology, School of Medicine and Health Sciences, The George Washington University, Washington, DC 20052

[4]Department of Neurobiology and Kavli Institute for Neuroscience, School of Medicine, Yale University, New Haven, CT 06510

*To whom correspondence should be addressed:

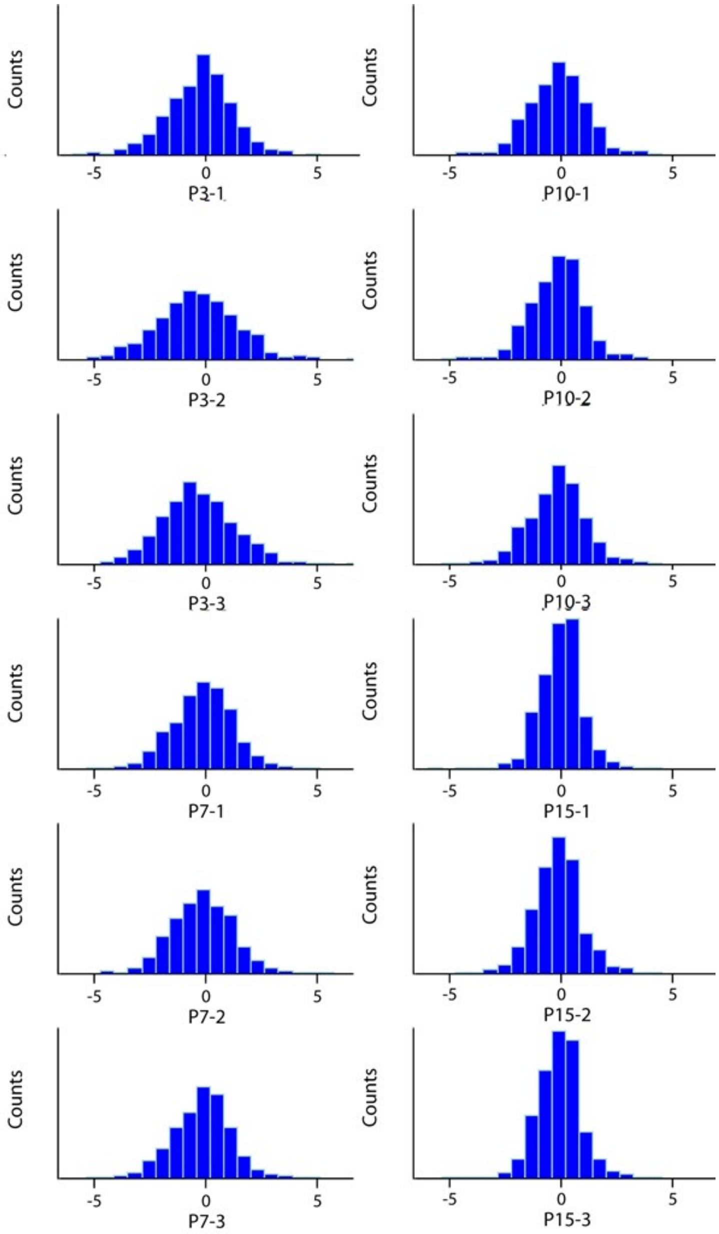

Figure S1. Distribution of the protein expression in the postnatal CC.

The density plot of log2 transformed SILAC ratio for all identified proteins from each sample.

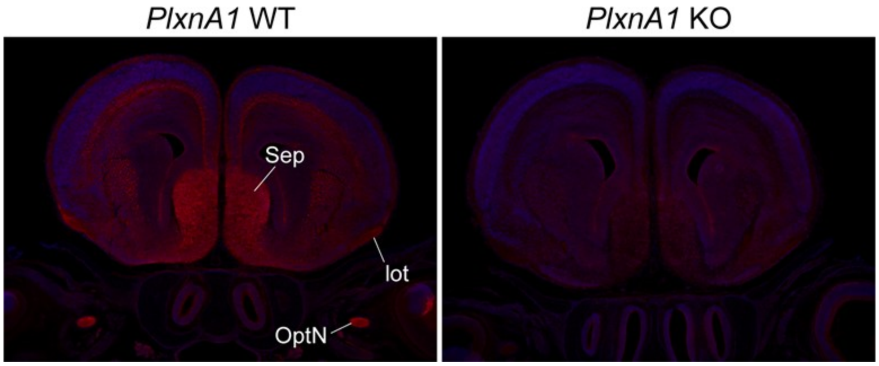

Figure S2. Confirmation of the specificity of anti-PlxnA1 antibody.

Immunohistochemistry using rabbit polyclonal anti-PlxnA1 antibody on brain sections of *PlxnA1* wild-type (WT) and knockout (KO) mice (red). The sections were counterstained with DAPI (blue). Specific labeling is observed in several regions in the WT tissue including the cortical white matter, septum (Sep), lateral olfactory tract (lot) and optic nerve (OptN). No labeling is observed in the KO tissue.

Table S1. Proteome of postnatal CC samples.

The values represent log2 transformed SILAC ratios.

Table S2. K-means clustering of proteins in the developing CC.

Table S3. Functional annotations of proteins in each cluster.

Table S4. List of common and unique GO terms between clusters.

Table S5. Functional annotation clustering of proteins in each cluster.
